# Screening for Maternal Thyroid Dysfunction in Pregnancy: A Review of the Clinical Evidence and Current Guidelines

**DOI:** 10.1155/2013/851326

**Published:** 2013-05-20

**Authors:** Donny L. F. Chang, Elizabeth N. Pearce

**Affiliations:** Section of Endocrinology, Diabetes and Nutrition, Boston University School of Medicine and Boston Medical Center, 88 East Newton Street, Evans 201, Boston, MA 02118, USA

## Abstract

Observational studies have demonstrated that maternal thyroid dysfunction and thyroid autoimmunity in pregnancy may be associated with adverse obstetric and fetal outcomes. Treatment of overt maternal hyperthyroidism and overt hypothyroidism clearly improves outcomes. To date there is limited evidence that levothyroxine treatment of pregnant women with subclinical hypothyroidism, isolated hypothyroxinemia, or thyroid autoimmunity is beneficial. Therefore, there is ongoing debate regarding the need for universal screening for thyroid dysfunction during pregnancy. Current guidelines differ; some recommend an aggressive case-finding approach, whereas others advocate testing only symptomatic women or those with a personal history of thyroid disease or other associated medical conditions.

## 1. Introduction

It is well documented that maternal thyroid dysfunction is associated with adverse outcomes in the mother and fetus, including miscarriage, preterm delivery, eclampsia, pre-eclampsia, and placental abruption [1–6]. Decreased availability of maternal thyroid hormone may also impair neurological development of the fetus as several studies have reported decreased IQ in infants born to mothers with either overt hypothyroidism (OH) [7], hypothyroxinemia [8], or thyroid peroxidase antibody (TPO Ab) positivity [9]. However, there is limited and conflicting evidence regarding the impact of intervention on improving health outcomes in pregnant women with subclinical hypothyroidism (SH) and with euthyroid autoimmune disease in pregnancy.

There is ongoing debate regarding the need for universal screening for thyroid dysfunction during pregnancy. Current guidelines differ between an aggressive case finding approach [10–13] versus testing only symptomatic women or those with a personal history of thyroid disease or other associated medical condition [14, 15] (Box 1). The differing expert opinions are likely due to the fact that the majority of the evidence regarding thyroid dysfunction in pregnancy is based on observational studies. There is a paucity of large, prospective, randomized controlled trials demonstrating treatment benefits.

This review examines the current clinical evidence as it relates to screening for thyroid dysfunction and euthyroid autoimmune disease in pregnancy. We also compare the different management guidelines currently available, and how these guidelines have influenced clinical practice to date. We have referenced the current US guidelines along with The Cochrane Collaboration for this review. There may be other regional guidelines which we have not cited.

## 2. Thyroid Function Testing in Pregnancy

Normal pregnancy is associated with significant changes in maternal thyroid physiology. Serum thyroid-stimulating hormone (TSH) concentration is the initial and most reliable test for assessing thyroid function in pregnancy [16]. Serum TSH testing is relatively inexpensive, readily available, and is a reliable test in pregnancy, assuming that trimester-specific reference ranges are applied. A decline in TSH levels in the first trimester is seen due to elevation of human chorionic gonadotropin (hCG), which functions as a weak stimulator of the TSH receptor. Due to these dynamic changes during pregnancy, use of trimester-specific and assay-specific TSH normal ranges is recommended. Where such reference ranges are not available, the following cutoffs may be used: first trimester, <2.5 mIU/L; second trimester, <3 mIU/L; third trimester, <3 mIU/L [11, 12]. 

Thyroid function tests during pregnancy are also affected by estrogen-mediated increases in the level of thyroxine-binding globulins (TBG). Total T3 and T4 levels increase starting in early pregnancy, due to the increased TBG levels, so that the upper limit of normal for total T3 and T4 in pregnancy is approximately 1.5-fold the upper limit of the nonpregnancy reference range. Free T4 assays may be unreliable in pregnancy due to interference by the high TBG levels [17, 18]. Method-specific and trimester-specific reference ranges for direct immunoassays of free T4 are not currently widely available. The free T4 index may be more reliable than free T4 assays during pregnancy, and its use is advocated by the 2012 Endocrine Society guidelines [11, 17].

## 3. Hyperthyroidism

Overt hyperthyroidism occurs in approximately 0.1–0.4% of pregnancies [19] and is defined as a serum TSH level below the trimester-specific reference range with elevated levels of free T3, free T4 or both. The most common cause of overt hyperthyroidism in pregnancy is Graves' disease. Other causes include gestational transient thyrotoxicosis, toxic adenoma or multinodular goiter, thyroiditis, or excessive hormone intake. 

Definitive diagnosis can be more challenging in pregnancy since radioactive iodine thyroid scans are contraindicated. However, correlation with signs and symptoms may help elucidate the diagnosis. A diffuse goiter, ophthalmopathy, hyperthyroid symptoms prior to pregnancy, and serum thyroid hormone receptor antibody (TRAb) positivity favor the diagnosis of Graves' disease. Transient gestational thyrotoxicosis is more common in women with morning sickness, especially those with the most severe form, hyperemesis gravidarum [20]. 

Pregnant women with untreated overt hyperthyroidism are at increased risk for spontaneous miscarriage, congestive heart failure, thyroid storm, preterm birth, pre-eclampsia, fetal growth restriction, and increased perinatal morbidity and mortality [21–23]. Treatment of overt Graves' hyperthyroidism in pregnancy to achieve adequate metabolic control has been associated with improved pregnancy outcomes [24]. 

Subclinical hyperthyroidism is defined as a serum TSH level below the trimester-specific reference range with normal levels of free T3 and T4. Although various TSH cut-off values have been used in studies to define subclinical hyperthyroidism, in general, subclinical maternal hyperthyroidism has not been found to be associated with adverse maternal or fetal outcomes [5], and recommendations are for monitoring in pregnancy, but not therapy [11, 12]. 

## 4. Overt Hypothyroidism

Around 0.5% of all pregnant women will have overt hypothyroidism (OH) [3, 25], defined as an elevated TSH level with a decreased level of free T4 [5, 26, 27]. The most common etiology of OH in pregnant women is chronic autoimmune thyroiditis (Hashimoto's thyroiditis). Other causes of OH include endemic iodine deficiency (ID), and prior radioactive iodine therapy or thyroidectomy. 

While severe endemic ID can lead to OH, mild-to-moderate ID is more frequently associated with isolated hypothyroxinemia rather than OH. Currently, 30 countries worldwide are considered iodine deficient [28]. Although median urinary iodine concentrations and other measures can be used to determine the iodine status of populations, there are no biomarkers to diagnose ID in individuals [29, 30].

Untreated OH in pregnancy has consistently been shown to be associated with an increased risk for adverse pregnancy complications, as well as detrimental effects on fetal neurocognitive development [7]. Specific adverse outcomes associated with maternal OH include increased risks for premature birth, low birth weight, and miscarriage [1]. Allan et al. and Abalovich et al. demonstrated an increased risk of fetal loss in patients with OH [3, 4]. Leung et al. demonstrated a 22% risk of gestational hypertension in pregnant women with OH, higher than for euthyroid women or those with subclinical hypothyroidism (SH) [2].

Because of the clear associations between OH and risk to the mother and fetus, treatment of overt hypothyroidism during pregnancy is mandatory [10–12]. The goal of levothyroxine (LT4) treatment is to normalize maternal serum TSH values to within the trimester-specific pregnancy reference range [10–12].

## 5. Subclinical Hypothyroidism (SH)

SH is defined as an elevated TSH level with a normal level of circulating free T4. The prevalence of SH during pregnancy in the US is estimated to be 0.25–2.5% [3, 25]. Symptoms of SH, if present, are typically subtle, and might be attributed to pregnancy. Even in OH individuals there can be a major discrepancy between symptoms and thyroid status. Canaris et al. demonstrated that although OH patients were more likely to report hypothyroid symptoms than euthyroid individuals, only 30% of OH patients were symptomatic whereas 17% of controls reported hypothyroid symptoms [31]. In addition, close to 20% of OH patients reported no symptoms at all. Thus, while the presence of symptoms may be suggestive of either OH or SH, their absence fails to exclude it. 

SH in pregnancy has been associated with adverse maternal outcomes in observational studies including eclampsia, pre-eclampsia, placental abnormalities, miscarriages, pre-term labor, and low birth weights [1–5]. However, other studies have not found adverse obstetrical outcomes in subclinically hypothyroid pregnant women [32, 33] and adverse outcomes have varied from study to study.

A single prospective randomized control trial (RCT) to date has assessed the effect of LT4 therapy for mild maternal thyroid failure during pregnancy on offspring IQ [34]. Lazarus et al. randomized mildly hypothyroid or hypothyroxinemic pregnant women to LT4 treatment versus no treatment. At age 3, children of women treated with LT4 (started at a median gestational age of 13 weeks) had IQ tests which did not differ from the children of untreated women. These results have been criticized on the basis that intervention began in many women following the first trimester, which is the critical time for fetal brain development. Furthermore, IQ testing may not be the most sensitive method of assessing the effect of hypothyroidism on neural development in 3 year olds. Another ongoing large scale prospective randomized controlled trial sponsored by the National Institute of Child Health and Human Development is screening pregnant women less than 20 weeks gestation for SH or hypothyroxinemia, and randomizing to treatment with LT4 or placebo until delivery. The offspring will have annual developmental testing until age 5 to determine whether therapy is effective in improving IQ [35]. 

Treatment of SH is not universally advocated as there are limited data demonstrating a beneficial effect of thyroid hormone therapy on health outcomes [11, 12]. One RCT by Negro et al. has shown that treatment decreases the occurrence of adverse events in the mother and fetus [36]. The objective of the study was to determine if treatment of thyroid disease during pregnancy decreased the incidence of adverse outcomes and to compare the efficacy of universal screening versus case finding in detecting thyroid dysfunction. Whereas universal screening did not result in a decrease in adverse outcomes, treatment of thyroid abnormalities identified by screening a low-risk group resulted in a significant decrease in adverse outcomes. 

Based on the Negro et al. study, the American Thyroid Association (ATA) recommended that women with SH who are TPO Ab positive should be treated with LT4 [12]. The ATA felt there was not enough evidence to recommend for or against LT4 treatment for TPO Ab negative women with SH and TSH <10 mIU/L. However, the joint American Association of Clinical Endocrinologists (AACE)/ATA and The Endocrine Society (TES) guidelines recommend LT4 replacement in all women with SH (TSH >2.5 mIU/L in 1st trimester) regardless of TPO Ab status [10].

## 6. Antithyroid Antibody Positivity in Pregnancy

TPO and thyroglobulin (TG) autoantibodies can be detected in 10–20% of women of childbearing age [37]. The majority of women who test positive for thyroid autoantibodies are euthyroid. Sixteen percent of the women who are euthyroid and positive for TPO or TG antibodies in the first trimester will develop a TSH that exceeds 4.0 mIU/L by the third trimester [38], and 33%–50% of women who are positive for TPO or TG antibody in the first trimester will develop postpartum thyroiditis [39–41]. 

Thyroid autoimmunity in pregnancy has been associated with adverse pregnancy outcomes, including miscarriage [42–48], recurrent abortion [49–55], preterm births [37, 48, 56, 57], and low IQ [8]. 

In a prospective study published in 1990, the miscarriage rate was doubled in euthyroid women who tested positive for thyroid autoantibodies compared with women who tested negative [42]. The increased rate of miscarriage was not related to demographic variables nor to the presence of cardiolipin antibodies. A recent meta-analysis demonstrated an association between thyroid autoantibodies and spontaneous miscarriage, as well as preterm births [37]. A single RCT evaluated the effect of treatment of euthyroid TPO Ab positive pregnant women on miscarriage and showed a significant reduction in miscarriage rates, as well as the rates of preterm births [48]. There is currently an ongoing large, multicenter, double blind, placebo-controlled RCT in the United Kingdom to determine the efficacy of low dose LT4 treatment for obstetric and neonatal outcomes in women with thyroid antibodies (TABLET trial) [58]. 

The data for an association between thyroid antibodies and recurrent pregnancy loss are less robust than for sporadic loss and somewhat contradictory. Recurrent miscarriages can be due to many potential causes, and endocrine dysfunction may only account for 15%–20% of all cases. In addition, many of the previous trials did not control for other potential causes of recurrent losses. Therefore, none of the guidelines recommend universal TPO Ab testing or treatment of euthyroid TPO Ab positive women [10–12]. 

## 7. Isolated Hypothyroxinemia

Isolated hypothyroxinemia is defined as a normal maternal TSH concentration with FT4 concentrations in the lower 5th or 10th percentiles of the reference range. Observational studies to date have not shown any adverse obstetric outcomes [32, 59]. However, it is unclear whether isolated maternal hypothyroxinemia is associated with adverse neurodevelopmental outcomes. Pop et al. reported a decrease in psychomotor test scores among offspring born to women with FT4 indices in the lowest 10th percentile [60]. Henrichs et al. observed a similar reduction in the IQ of the offspring of mothers with isolated hypothyroxinemia during the first trimester [61]. Recent data from The Netherlands showed that children born to women with isolated hypothyroxinemia had a 1.5- to 2-fold increased risk of cognitive delays in early childhood [62]. The Controlled Antenatal Thyroid Study did not demonstrate a benefit of treatment in this subgroup; however, conclusions were limited due to the small numbers of women and the post-hoc nature of the analysis [34]. Other studies have not found a correlation between maternal thyroid hypofunction in pregnancy and offspring neurodevelopment [63–65].

Although there are limited data to suggest harm from isolated hypothyroxinemia, no interventional data to date has shown improvement with LT4 therapy. Thus, the ATA 2012 guidelines do not recommend routine screening or treatment for isolated hypothyroxinemia in pregnancy [12]. The Endocrine Society 2012 guidelines do not comment on isolated hypothyroxinemia in pregnancy [10].

## 8. Screening for Thyroid Dysfunction

Given that most thyroid dysfunction that occurs in pregnant women is SH, and given the lack of clear data for efficacy of treatment, there is ongoing debate regarding the need for universal screening for thyroid dysfunction during pregnancy versus a case-finding approach [10–13] versus testing only symptomatic women or those with personal history of thyroid disease or other associated medical conditions [14, 15] (Box 1).

Appropriately dosed LT4 therapy in pregnancy does not confer any risks for the mother or fetus. However, the benefits of treating SH are unclear based on current published RCTs. The potential risks of universal screening would be: (1) costs of treatment, followup, and monitoring, (2) possible misinterpretation of TFTs resulting in inappropriate treatment, and (3) inappropriately dosed LT4 treatment for SH women, resulting in over- or undertreatment. Experienced caregivers should be involved in interpretation of TFTs in pregnancy to avoid misdiagnoses and initiation of inappropriate treatments (e.g., misinterpreting a low TSH as abnormal and inappropriately initiating treatment for hyperthyroidism). If patients are started on LT4 therapy, they need close monitoring to ensure euthyroidism. In a survey of a non-pregnant population, Canaris et al. demonstrated that over 40 percent of patients taking thyroid medications were not at target range and were either hypo- or hyperthyroid [66].

The most recent practice guidelines from the American College of Obstetricians and Gynecology (ACOG) in 2007 recommend thyroid testing only in high-risk pregnant women who are symptomatic, or have a personal history of thyroid disorders, personal history of type I diabetes or other autoimmune disorders [15]. The 2007 ACOG guidelines specifically do not recommend testing in asymptomatic women or women with small goiters. The Society for Maternal-Fetal Medicine supports the recommendations of ACOG [14]. 

The American Thyroid Association (ATA) in 2011 recommended against universal screening of healthy women for thyroid dysfunction during pregnancy [12]. However, a case-finding approach was advocated to identify individuals at high risk for hypothyroidism (see Box 2). In guidelines published in 2012, the Endocrine Society Task Force could not reach agreement on thyroid testing recommendations for pregnant women [11]. Some members recommended screening of all pregnant women for serum TSH abnormalities by the 9th week or at the time of their first visit. Others recommended against universal screening of pregnant women at the time of their first visit and instead supported aggressive case finding to identify high-risk women. This case-finding approach is similar to the 2011 ATA guidelines (see Box 2). The American Association of Clinical Endocrinologists (AACE)/ATA Hypothyroidism guidelines also currently recommend a case-finding approach [10]. 

## 9. Efficacy of Case Finding

Recent studies have examined the efficacy of the previous Endocrine Society 2007 guidelines' targeted case-finding approach in identifying women with thyroid dysfunction during early pregnancy [67]. Vaidya et al. showed that among a cohort of pregnant women, serum TSH levels were >4.2 mIU/L in 2.6% of women and the prevalence was higher in the high-risk group versus the low-risk group [68]. However, 30% of the women with an elevated TSH were in the low-risk population, suggesting that the 2007 case-finding guidelines [67] would miss about one third of pregnant women with subclinical and overt hypothyroidism. In another study, Horacek et al. estimated that 55% of women with thyroid abnormalities (including positive thyroid antibody test results and hypothyroxinemia as well as subclinical and overt hypothyroidism) would have been missed using a case-finding approach rather than a universal screening approach [69]. In a retrospective cohort study performed in 2011, we reported that among 983 consecutive pregnant women in Boston, up to 80% of women with elevated TSH levels might have been missed using a case-finding approach rather than universal screening [70]. 

It is important to note that the previous studies were based on a slightly less comprehensive case-finding approach from the Endocrine Society 2007 [67] compared to the newer guidelines from the Endocrine Society in 2012 [11] and the ATA in 2011 [12]. Studies are needed to determine how effective the new case-finding criteria are at detecting overt and subclinical hypothyroidism in pregnancy. 

## 10. Thyroid Pregnancy Screening in Current Clinical Practice

Conflicting guidelines can lead to variability in testing rates among practitioners and across regions [71, 72]. A retrospective national US study which included 502,036 pregnant women reported that 23% were tested for gestational hypothyroidism, of whom 15.5% had elevated serum TSH values [73]. In 2009, Haymart conducted a survey of Obstetricians and Family Medicine practitioners from Wisconsin and showed that only 11.5% of providers had read the Endocrine Society 2007 guidelines, but that reading the guidelines was associated with increased likelihood of screening for thyroid disease risk [72]. In 2010, Vaidya et al. surveyed members of the European Thyroid Association; 42% of respondents reported screening all pregnant women for hypothyroidism, 43% reported that they used targeted case finding, and 17% of respondents did not perform routine thyroid testing [74]. Our 2011 Boston study at a single academic center showed that there was a high rate of screening (84.6%) [70].

## 11. Cost-Effectiveness Studies

A recent study comparing ACOG 2002 guidelines (screening only symptomatic women or those with history of thyroid or associated diseases) versus universal screening for SH in pregnancy demonstrated that universal screening was by far the most cost-effective strategy under a wide range of circumstances [75]. Using a decision analysis model, Thung et al. determined that universally screening 100,000 women can result in over $8,000,000 in cost savings and improve offspring outcome by reducing the number of offspring with cognitive impairment. The cost-savings were determined from the relatively low cost of thyroid screening tests and treatment of SH compared to the relatively large additional lifetime costs incurred by individuals with neurodevelopmental impairment. However, a benefit of treatment on offspring neurodevelopment has not to date been demonstrated in interventional studies [34]. 

Another recent study by Dosiou et al. also supports universal screening of pregnant women as a cost-effective measure in various clinical scenarios [76]. They developed a model in which women were screened in the first trimester of pregnancy with TSH and TPOA Ab. Women with TSH elevations underwent further testing, and treatment with LT4 was initiated when indicated. Their analysis showed that universal screening of pregnant women was a cost-effective measure compared to no screening at all, but also when compared to screening of high-risk women alone. Importantly, universal screening remained cost-effective even when only OH, rather than SH, was detected and treated. 

## 12. Conclusions

The issue of universal screening for thyroid dysfunction and euthyroid autoimmune disease in pregnancy remains controversial. Observational studies have demonstrated adverse maternal and fetal outcomes in both women with subclinical hypothyroidism and in euthyroid women who test positive for thyroid autoantibodies. To date, there is limited evidence to demonstrate that LT4 treatment can improve outcomes.

In our personal practice, we perform screening TSH during pregnancy, and treat all pregnant patients with SH. However, we cannot recommend universal screening at this time given the paucity of interventional studies. 

As more data become available regarding the effectiveness of treatment and screening for thyroid dysfunction in pregnant women, recommendations for thyroid testing in pregnancy and clinical practice patterns will likely become more uniform.

## Figures and Tables

**Box 1:**
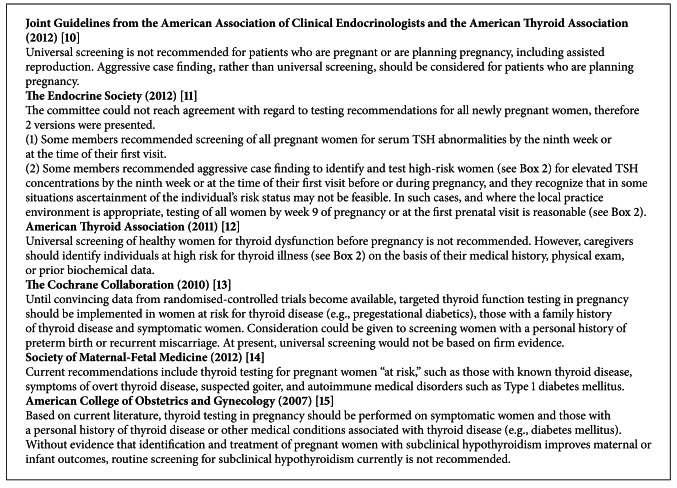
Screening recommendations for thyroid disorders in pregnancy.

**Box 2:**
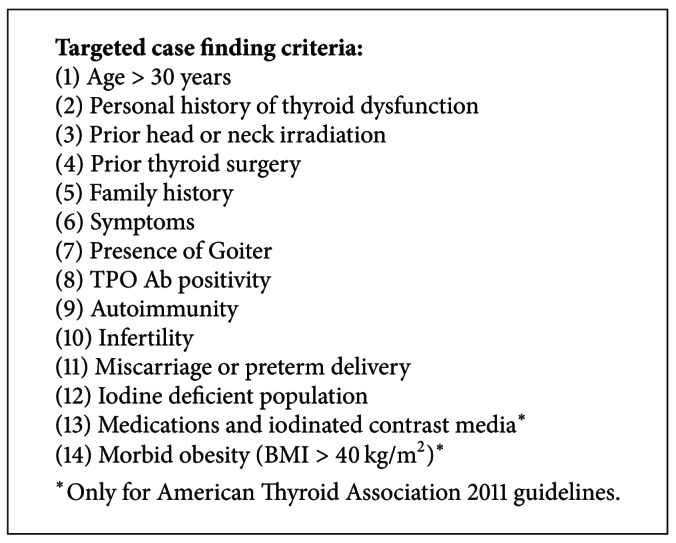
Case-finding approach for high risk patients based on The Endocrine Society 2012 [11] and American Thyroid Association 2011 [12] guidelines.

## References

[1] Davis LE, Leveno KJ, Cunningham FG (1988). Hypothyroidism complicating pregnancy. *Obstetrics and Gynecology*.

[2] Leung AS, Millar LK, Koonings PP, Montoro M, Mestman JH (1993). Perinatal outcome in hypothyroid pregnancies. *Obstetrics and Gynecology*.

[3] Allan WC, Haddow JE, Palomaki GE (2000). Maternal thyroid deficiency and pregnancy complications: implications for population screening. *Journal of Medical Screening*.

[4] Abalovich M, Gutierrez S, Alcaraz G, Maccallini G, Garcia A, Levalle O (2002). Overt and subclinical hypothyroidism complicating pregnancy. *Thyroid*.

[5] Casey BM, Dashe JS, Wells CE (2005). Subclinical hypothyroidism and pregnancy outcomes. *Obstetrics and Gynecology*.

[6] Ashoor G, Maiz N, Rotas M, Jawdat F, Nicolaides KH (2010). Maternal thyroid function at 11 to 13 weeks of gestation and subsequent fetal death. *Thyroid*.

[7] Haddow JE, Palomaki GE, Allan WC (1999). Maternal thyroid deficiency during pregnancy and subsequent neuropsychological development of the child. *The New England Journal of Medicine*.

[8] Pop VJ, Kuijpens JL, van Baar AL (1999). Low maternal free thyroxine concentrations during early pregnancy are associated with impaired psychomotor development in infancy. *Clinical Endocrinology*.

[9] Pop VJ, de Vries E, van Baar AL (1995). Maternal thyroid peroxidase antibodies during pregnancy: a marker of impaired child development?. *Journal of Clinical Endocrinology and Metabolism*.

[10] Garber JR, Cobin RH, Gharib H (2012). Clinical practice guidelines for hypothyroidism in adults: co-sponsored by American Association of Clinical Endocrinologists and the American Thyroid Association. *Endocrine Practice*.

[11] De Groot L, Abalovich M, Alexander EK (2012). Management of thyroid dysfunction during pregnancy and postpartum: an Endocrine Society clinical practice guideline. *Journal of Clinical Endocrinology and Metabolism*.

[12] Stagnaro-Green A, Abalovich M, Alexander E (2011). Guidelines of the American Thyroid Association for the diagnosis and management of thyroid disease during pregnancy and postpartum. *Thyroid*.

[13] Reid SM, Middleton P, Cossich MC, Crowther CA (2010). Interventions for clinical and subclinical hypothyroidism in pregnancy. *Cochrane Database of Systematic Reviews*.

[14] Society for Maternal-Fetal Medicine (SMFM), Gyamfi-Bannerman C (2012). Screening for thyroid disease during pregnancy. *Contemporary OB/Gyn*.

[15] Committee on Patient Safety and Quality Improvement (2007). ACOG Committee Opinion No. 381: subclinical hypothyroidism in pregnancy. *Obstetrics and Gynecology*.

[16] Glinoer D, Spencer CA (2010). Serum TSH determinations in pregnancy: how, when and why?. *Nature Reviews Endocrinology*.

[17] Lee RH, Spencer CA, Mestman JH (2009). Free T4 immunoassays are flawed during pregnancy. *American Journal of Obstetrics and Gynecology*.

[18] Sapin R, D’Herbomez M, Schlienger JL (2004). Free thyroxine measured with equilibrium dialysis and nine immunoassays decreases in late pregnancy. *Clinical Laboratory*.

[19] Glinoer D (1998). Thyroid hyperfunction during pregnancy. *Thyroid*.

[20] Goodwin TM, Montoro M, Mestman JH (1992). Transient hyperthyroidism and hyperemesis gravidarum: clinical aspects. *American Journal of Obstetrics and Gynecology*.

[21] Davis LE, Lucas MJ, Hankins GDV, Roark ML, Cunningham FG (1989). Thyrotoxicosis complicating pregnancy. *American Journal of Obstetrics and Gynecology*.

[22] Millar LK, Wing DA, Leung AS, Koonings PP, Montoro MN, Mestman JH (1994). Low birth weight and preeclampsia in pregnancies complicated by hyperthyroidism. *Obstetrics and Gynecology*.

[23] Kriplani A, Buckshee K, Bhargava VL, Takker D, Ammini AC (1994). Maternal and perinatal outcome in thyrotoxicosis complicating. *European Journal of Obstetrics Gynecology and Reproductive Biology*.

[24] Momotani N, Noh J, Oyanagi H (1986). Antithyroid drug therapy for Graves' disease during pregnancy: optimal regimen for fetal thyroid status. *The New England Journal of Medicine*.

[25] Klein RZ, Haddow JE, Faix JD (1991). Prevalence of thyroid deficiency in pregnant women. *Clinical Endocrinology*.

[26] Stagnaro-Green A, Roman SH, Cobin RH, El-Harazy E, Wallenstein S, Davies TF (1992). A prospective study of lymphocyte-initiated immunosuppression in normal pregnancy: evidence of a T-cell etiology for postpartum thyroid dysfunction. *Journal of Clinical Endocrinology and Metabolism*.

[27] Stagnaro-Green A (2011). Overt hyperthyroidism and hypothyroidism during pregnancy. *Clinical Obstetrics and Gynecology*.

[28] Pearce EN, Andersson M, Zimmermann M (2013). Global iodine nutrition—where do we stand in 2013?. *Thyroid*.

[29] WHO (2007). *Assessment of Iodine Deficiency Disorders and Monitoring Their Elimination. A Guide for Programme Managers*.

[30] Andersson M, Karumbunathan V, Zimmermann MB (2012). Global iodine status in 2011 and trends over the past decade. *Journal of Nutrition*.

[31] Canaris GJ, Steiner JF, Ridgway EC (1997). Do traditional symptoms of hypothyroidism correlate with biochemical disease?. *Journal of General Internal Medicine*.

[32] Cleary-Goldman J, Malone FD, Lambert-Messerlian G (2008). Maternal thyroid hypofunction and pregnancy outcome. *Obstetrics and Gynecology*.

[33] Männistö T, Vääräsmäki M, Pouta A (2009). Perinatal outcome of children born to mothers with thyroid dysfunction or antibodies: a prospective population-based cohort study. *Journal of Clinical Endocrinology and Metabolism*.

[34] Lazarus LH, Bestwick JP, Channon S (2012). Antenatal thyroid screening and childhood cognitive function. *The New England Journal of Medicine*.

[35] Eunice Kennedy Shriver National Institute of Child Health and Human Development (NICHD) Thyroid Therapy for Mild Thyroid Deficiency in Pregnancy (TSH). http://www.nhlbi.nih.gov/meetings/workshops/cardiorenal-hf-hd.htm.

[36] Negro R, Schwartz A, Gismondi R, Tinelli A, Mangieri T, Stagnaro-Green A (2010). Universal screening versus case finding for detection and treatment of thyroid hormonal dysfunction during pregnancy. *Journal of Clinical Endocrinology and Metabolism*.

[37] Thangaratinam S, Tan A, Knox E, Kilby MD, Franklyn J, Coomarasamy A (2011). Association between thyroid autoantibodies and miscarriage and preterm birth: Meta-analysis of evidence. *British Medical Journal*.

[38] Glinoer D, Riahi M, Grun JP, Kinthaert J (1994). Risk of subclinical hypothyroidism in pregnant women with asymptomatic autoimmune thyroid disorders. *Journal of Clinical Endocrinology and Metabolism*.

[39] Lazarus JH, Othman S (1991). Thyroid disease in relation to pregnancy. *Clinical Endocrinology*.

[40] Lazarus JH (1998). Prediction of postpartum thyroiditis. *European Journal of Endocrinology*.

[41] Weetman AP (1994). Prediction of post-partum thyroiditis. *Clinical Endocrinology*.

[42] Stagnaro-Green A, Roman SH, Cobin RH, El-Harazy E, Alvarez-Marfany A, Davies TF (1990). Detection of at-risk pregnancy by means of highly sensitive assays for thyroid autoantibodies. *Journal of the American Medical Association*.

[43] Glinoer D, Soto MF, Bourdoux P (1991). Pregnancy in patients with mild thyroid abnormalities: maternal and neonatal repercussions. *Journal of Clinical Endocrinology and Metabolism*.

[44] Lejeune B, Grun JP, De Nayer P, Servais G, Glinoer D (1993). Antithyroid antibodies underlying thyroid abnormalities and miscarriage or pregnancy induced hypertension. *British Journal of Obstetrics and Gynaecology*.

[45] Iijima T, Tada H, Hidaka Y, Mitsuda N, Murata Y, Amino N (1997). Effects of autoantibodies on the course of pregnancy and fetal growth. *Obstetrics and Gynecology*.

[46] Bagis T, Gokcel A, Saygili ES (2001). Autoimmune thyroid disease in pregnancy and the postpartum period: relationship to spontaneous abortion. *Thyroid*.

[47] Ghafoor F, Mansoor M, Malik T (2006). Role of thyroid peroxidase antibodies in the outcome of pregnancy. *Journal of the College of Physicians and Surgeons Pakistan*.

[48] Negro R, Formoso G, Mangieri T, Pezzarossa A, Dazzi D, Hassan H (2006). Levothyroxine treatment in euthyroid pregnant women with autoimmune thyroid disease: effects on obstetrical complications. *Journal of Clinical Endocrinology and Metabolism*.

[49] Pratt DE, Kaberlein G, Dudkiewicz A, Karande V, Gleicher N (1993). The association of antithyroid antibodies in euthyroid nonpregnant women with recurrent first trimester abortions in the next pregnancy. *Fertility and Sterility*.

[50] Pratt D, Novotny M, Kaberlein G, Dudkiewicz A, Gleicher N (1993). Antithyroid antibodies and the association with non-organ-specific antibodies in recurrent pregnancy loss. *American Journal of Obstetrics and Gynecology*.

[51] Bussen S, Steck T (1995). Thyroid autoantibodies in euthyroid non-pregnant women with recurrent spontaneous abortions. *Human Reproduction*.

[52] Bussen SS, Steck T (1997). Thyroid antibodies and their relation to antithrombin antibodies, anticardiolipin antibodies and lupus anticoagulant in women with recurrent spontaneous abortions (antithyroid, anticardiolipin and antithrombin autoantibodies and Lupus anticoagulant in habitual aborters). *European Journal of Obstetrics Gynecology and Reproductive Biology*.

[53] Kutteh WH, Yetman DL, Carr AC, Beck LA, Scott RT (1999). Increased prevalence of antithyroid antibodies identified in women with recurrent pregnancy loss but not in women undergoing assisted reproduction. *Fertility and Sterility*.

[54] Shoenfeld Y, Carp HJA, Molina V (2006). Autoantibodies and prediction of reproductive failure. *American Journal of Reproductive Immunology*.

[55] Iravani AT, Saeedi MM, Pakravesh J, Hamidi S, Abbasi M (2008). Thyroid autoimmunity and recurrent spontaneous abortion in Iran: a case-control study. *Endocrine Practice*.

[56] Stagnaro-Green A (2009). Maternal thyroid disease and preterm delivery. *Journal of Clinical Endocrinology and Metabolism*.

[57] Negro R (2011). Thyroid autoimmunity and pre-term delivery: brief review and meta-analysis. *Journal of Endocrinological Investigation*.

[58] Birmingham Clinical Trials Unit Randomised Controlled Trial of the Efficacy and Mechanism of Levothyroxine Treatment on Pregnancy and Neonatal Outcomes in Women with Thyroid Antibodies (TABLET). http://www.birmingham.ac.uk.

[59] Casey BM, Dashe JS, Spong CY, McIntire DD, Leveno KJ, Cunningham GF (2007). Perinatal significance of isolated maternal hypothyroxinemia identified in the first half of pregnancy. *Obstetrics and Gynecology*.

[60] Pop VJ, Brouwers EP, Vader HL, Vulsma T, van Baar AL, de Vijlder JJ (2003). Maternal hypothyroxinaemia during early pregnancy and subsequent child development: a 3-year follow-up study. *Clinical Endocrinology*.

[61] Henrichs J, Bongers-Schokking JJ, Schenk JJ (2010). Maternal thyroid function during early pregnancy and cognitive functioning in early childhood: the Generation R study. *Journal of Clinical Endocrinology and Metabolism*.

[62] Li Y, Shan Z, Teng W (2010). Abnormalities of maternal thyroid function during pregnancy affect neuropsychological development of their children at 25–30 months. *Clinical Endocrinology*.

[63] Oken E, Braverman LE, Platek D, Mitchell ML, Lee SL, Pearce EN (2009). Neonatal thyroxine, maternal thyroid function, and child cognition. *Journal of Clinical Endocrinology and Metabolism*.

[64] Chevrier J, Harley KG, Kogut K, Holland N, Johnson C, Eskenazi B (2011). Maternal thyroid function during the second half ofpregnancy and child neurodevelopment at 6, 12, 24, and 60 months of age. *Journal of Thyroid Research*.

[65] Julvez J, Alvarez-Pedrerol M, Rebagliato M (2013). Thyroxine levels during pregnancy in healthy women and early child neurodevelopment. *Epidemiology*.

[66] Canaris GJ, Manowitz NR, Mayor G, Ridgway EC (2000). The Colorado thyroid disease prevalence study. *Archives of Internal Medicine*.

[67] Abalovich M, Amino N, Barbour LA (2007). Clinical practice guideline: management of thyroid dysfunction during pregnancy and postpartum: an endocrine society clinical practice guideline. *Journal of Clinical Endocrinology and Metabolism*.

[68] Vaidya B, Anthony S, Bilous M (2007). Brief report: detection of thyroid dysfunction in early pregnancy: universal screening or targeted high-risk case finding?. *Journal of Clinical Endocrinology and Metabolism*.

[69] Horacek J, Spitalnikova S, Dlabalova B (2010). Universal screening detects two-times more thyroid disorders in early pregnancy than targeted high-risk case finding. *European Journal of Endocrinology*.

[70] Chang DL, Leung AM, Braverman LE, Pearce EN (2011). Thyroid testing during pregnancy at an Academic Boston area medical center. *Journal of Clinical Endocrinology and Metabolism*.

[71] Haddow JE, McClain MR, Palomaki GE, Kloza EM, Williams J (2006). Screening for thyroid disorders during pregnancy: results of a survey in Maine. *American Journal of Obstetrics and Gynecology*.

[72] Haymart MR (2010). The role of clinical guidelines in patient care: thyroid hormone replacement in women of reproductive age. *Thyroid*.

[73] Blatt/surname AJ, Nakamoto JM, Kaufman HW (2012). National status of testing for hypothyroidism during pregnancy and postpartum. *Journal of Clinical Endocrinology and Metabolism*.

[74] Vaidya B, Hubalewska-Dydejczyk A, Laurberg P, Negro R, Vermiglio F, Poppe K (2012). Treatment and screening of hypothyroidism in pregnancy: results of a European survey. *European Journal of Endocrinology*.

[75] Thung SF, Funai EF, Grobman WA (2009). The cost-effectiveness of universal screening in pregnancy for subclinical hypothyroidism. *American Journal of Obstetrics and Gynecology*.

[76] Dosiou C, Barnes J, Schwartz A, Negro R, Crapo L, Stagnaro-Green A (2012). Cost-effectiveness of universal and risk-based screening for autoimmune thyroid disease in pregnant women. *Journal of Clinical Endocrinology and Metabolism*.

